# Corticospinal Excitability Is Modulated as a Function of Postural Perturbation Predictability

**DOI:** 10.3389/fnhum.2018.00068

**Published:** 2018-02-27

**Authors:** Kimiya Fujio, Hiroki Obata, Taku Kitamura, Noritaka Kawashima, Kimitaka Nakazawa

**Affiliations:** ^1^Department of Rehabilitation Science, Faculty of Health Care Science, Chiba Prefectural University of Health Sciences, Chiba, Japan; ^2^Department of Rehabilitation for Movement Functions, Research Institute of National Rehabilitation Center for Persons with Disabilities, Saitama, Japan; ^3^Department of Humanities and Social Sciences, Institute of Liberal Arts, Kyushu Institute of Technology, Fukuoka, Japan; ^4^Sports Science Laboratory, Department of Life Sciences, Graduate School of Arts and Sciences, University of Tokyo, Tokyo, Japan

**Keywords:** posture, corticospinal pathway, prediction, tibialis anterior muscle, transcranial magnetic stimulation

## Abstract

Recent studies demonstrated that the corticospinal pathway is one of the key nodes for the feedback control of human standing and that the excitability is flexibly changed according to the current state of posture. However, it has been unclear whether this pathway is also involved in a predictive control of human standing. Here, we investigated whether the corticospinal excitability of the soleus (SOL) and tibialis anterior (TA) muscles during standing would be modulated anticipatorily when perturbation was impending. We measured the motor-evoked potential (MEP) induced by transcranial magnetic stimulation over the motor cortex at six stimulus intensities. Three experimental conditions were set depending on predictabilities about perturbation occurrence and onset: No perturbation, No Cue, and Cue conditions. In the Cue condition, an acoustic signal was given as timing information of perturbation. The slope of the stimulus–response relation curve revealed that the TA-MEP was enhanced when postural perturbation was expected compared to when the perturbation was not expected (No Perturbation vs. No Cue, 0.023 ± 0.004 vs. 0.042 ± 0.007; No Perturbation vs. Cue, 0.023 ± 0.004 vs. 0.050 ± 0.009; Bonferroni correction, *p* = 0.01, respectively). In addition, two-way analysis of variance (intensity × condition) revealed the main effect of condition (*F*_(1,13)_ = 6.31, *p* = 0.03) but not intensity and interaction when the MEP amplitude of the Cue and No Cue conditions was normalized by that in No Perturbation, suggesting the enhancement more apparent when timing information was given. The SOL-MEP was not modulated even when perturbation was expected, but it slightly reduced due to the timing information. The results of an additional experiment confirmed that the acoustic cue by itself did not affect the TA- and SOL-MEPs. Our findings suggest that a prediction of a future state of standing balance modulates the corticospinal excitability in the TA, and that the additional timing information facilitates this modulation. The corticospinal pathway thus appears to be involved in mechanisms of the predictive control as well as feedback control of standing posture.

## Introduction

It has been established that the corticospinal pathway is involved in the regulation of human bipedal standing. Several studies using transcranial magnetic stimulation (TMS) have demonstrated that the excitability of this pathway is modulated according to postural demands. The excitability of the corticospinal pathways in the tibialis anterior (TA) and soleus (SOL) muscles was enhanced while the subjects stood on an unstable rocking plate (Solopova et al., 2003), whereas it was decreased during supported standing (Tokuno et al., 2009). These findings suggest that the contribution of the corticospinal pathway increases when standing posture is exposed to unstable conditions.

Postural perturbation is thought to be one cause of unstable posture. Given that the excitability of the corticospinal pathways is enhanced under unstable conditions, we have speculated that the excitability would also be enhanced when a postural perturbation is predicted. However, it has not been known how the excitability would be modulated in response to an upcoming perturbation. There has been some research regarding the modulation of the long-latency electromyography (EMG) response with predictions to postural perturbation (Horak et al., 1989). Since the long-latency EMG response is mediated by the transcortical pathway (Petersen et al., 1998; Taube et al., 2006), the effects of prediction on the corticospinal pathway may be inferred in part based on the long-latency EMG response.

The long-latency EMG responses of the ankle plantar muscle and dorsiflexor muscle were reported to be enhanced when the posture was unstable (Christensen et al., 2001; Nakazawa et al., 2003). It was also demonstrated that the amplitude of these EMG responses was markedly reduced when an advance notification about the timing of perturbation onset was given to the subjects (Nakazawa et al., 2009; Fujio et al., 2016). It is thus plausible that the excitability of a corticospinal pathway would be enhanced when a subject can predict that a postural perturbation is to occur, and the enhanced excitability could be reduced when the subject can predict the timing of the perturbation.

The purpose of this study was to clarify whether the excitability of the corticospinal pathways in the ankle plantar and flexor muscles would be modulated just before postural perturbation. To this end, we applied TMS on the leg area of the subjects’ contralateral motor cortex and examined how the predictabilities of the occurrence and the timing of perturbation influenced the corticospinal excitability in the preparatory period. We compared the corticospinal excitability in the TA and the SOL during normal standing to that before perturbation with and without an acoustic timing cue. In an additional experiment (Experiment 2), we further investigated whether the acoustic cue affected the corticospinal excitability, since it was reported that an auditory stimulation enhanced the corticospinal excitability through changes in the subject’s arousal (Lofberg et al., 2014). Here, we attempted to reveal whether the corticospinal pathway contributes to the predictive control of posture. These findings will have important implications for clinical settings, especially for the assessments of the risk of falling.

## Materials and Methods

### Study Participants

Fifteen healthy participants (age: 27.1 ± 1.7 years, all males) with no history of orthopedic, neurological, or cognitive disorders participated. All 15 participants participated in the first experiment (Experiment 1), and 6 of these participants (age: 30.2 ± 2.0 years) were recruited in the second experiment (Experiment 2). This study was carried out in accordance with the recommendations of the Ethics Review Committee for Experimental Research with Human Subjects of the Graduate School of Arts and Sciences, the University of Tokyo. All subjects gave written informed consent in accordance with the Declaration of Helsinki.

### Apparatus and Task

Postural perturbations were applied by a six-degrees-of-freedom motion platform system actuated by an electric servomotor (Motion base MB-150, Cosmate, Tokyo, Japan). We programed the platform motion to translate anteriorly in the horizontal plane underneath the participant’s stance (total displacement: 6.0 cm, peak velocity: 25.0 cm/s, peak acceleration 3.2 m/s^2^).

In both of the experiments described below, the participant stood on a force plate (Kyowa, Tokyo, Japan) attached to this movable platform. The participant was allowed to decide his initial foot position, and this position was traced on the force plate so that it was maintained constantly throughout the experiment. The center of pressure (COP) was calculated during a trial and was monitored on a display 1.5 m in front of the participant.

At the beginning of the experiment, the COP displacement during the participant’s normal standing was measured to determine a target COP position, defined as an averaged anteroposterior and mediolateral trajectory. The participant was instructed not to move his head or any limb intentionally and to aim for the COP target to try to maintain the same posture until the perturbation onset in all trials. We suspected that it is important to consistently apply the same perturbation and for the participants to avoid marked changes of posture trial by trial before the perturbation onset. The participants were required to keep their feet in place against perturbation.

The ground reaction forces for calculating the COP were recorded at a 4-kHz sampling frequency and low-pass-filtered at 10 Hz (fourth-order zero-lag Butterworth filter). Ten practice trials were performed to familiarize the participants with the perturbation. We controlled the time courses of the platform motion, the TMS, and the COP feedback by using LabVIEW software (National Instruments, Austin, TX, United States).

### EMG

Surface EMG activities were recorded from the TA and SOL muscles of the participants’ right leg. Bipolar electrodes (Ag-AgCl, 7 mm diameter) were placed 1.5 cm apart on the muscle belly of each muscle and wrapped with thin elastic bandages to hold the electrodes and lead lines stably. The EMG signals were amplified (MEG-6108 bioelectric amplifier, Nihon Kohden, Tokyo, Japan) with a bandpass filter (15–1000 Hz) and digitized at a sampling rate of 4 kHz. For the normalization of the EMG signals in each muscle, we obtained the M-max of the SOL and the TA by electrical stimulation (1-ms rectangular pulse) of the posterior tibial nerve and the common peroneal nerve, respectively.

For the stimulation of the posterior tibial nerve, the cathode was placed in the popliteal fossa, and the anode was placed on the patella. The common peroneal nerve was stimulated using bipolar surface electrodes below the neck of the fibula and the outer edge of the popliteal fossa. The intensity of stimulation was increased from the subthreshold level until the peak-to-peak amplitude of the M-response was increased no further.

### Transcranial Magnetic Stimulation (TMS)

Single-pulse TMS was delivered over the left motor cortex via a Magstim-200 stimulator (Magstim, Whitland, United Kingdom) with a 110-mm double cone coil. An experimenter carefully held the coil away from the back so as not to prevent his natural postural sway. After the detection of an optimal stimulus site where the largest TA-motor evoked potential (MEP) could be evoked, the motor threshold (×1.0 TA-MT) was determined while the participant was standing as the minimal intensity for eliciting the peak-to-peak amplitude in the TA exceeding 50 μV in 5 of 10 consecutive stimulations. The same site could also yield the MEPs of the SOL muscles in the standing posture. The same stimulus intensity and site as the TA were applied for the SOL, and the MEPs of both muscles were obtained by the identical single TMS pulse.

An optimal stimulation site on the scalp was marked with a felt pen to ensure consistent positioning of the coil over the hot spot. The coil position was also monitored online by a custom-built navigation system using a 3D motion capture system collected at 100 Hz (OptiTrack V100:R2, NaturalPoint, Corvallis, OR, United States). Three reflective markers each (for the subject’s head and the coil) were attached to determine the coil position relative to the participant’s head. An examiner referred to the marked position to guide the coil and adjust the direction in accord with the navigation system. All experiments were conducted by a single examiner who guided the coil position behind the participant and constantly supported the coil weight. The single-pulse TMS was delivered at 50 ms before the perturbation onset in all conditions.

### Experimental Conditions

We performed the following two experiments, carried out on separate days.

#### Experiment 1

**FIGURE 1:**
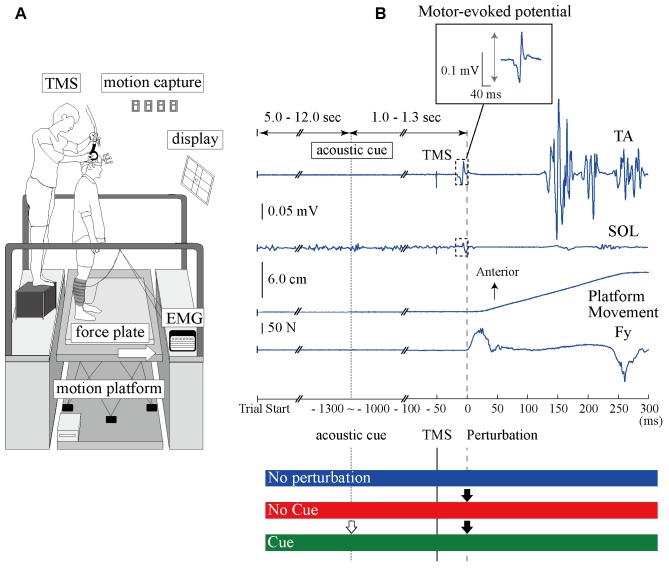
The experimental setup and the time course of TMS and anterior-surface translation in a single trial. **(A)** The devices and scene of the experiment. The site of the coil is guided using the 3D motion capture system. **(B)** Top, middle upper, middle lower, and bottom waveforms show the TA-EMG, SOL-EMG, displacement of the platform, and the force from the surface, respectively. Zero millisecond corresponds to the perturbation onset. The EMG response evoked at around 100 ms in the TA is more pronounced compared to the SOL response. The TMS is delivered 50 ms before the perturbation onset. The interesting range for analyzing the MEP amplitude is 40 ms after TMS onset. An acoustic cue is provided 1.0–1.3 ms before perturbation in the Cue condition as timing information. The BGA was calculated by the EMG activity from –50 to 0 ms. TA, tibialis anterior muscle; SOL, soleus muscle; Fy, net force in the anteroposterior component recorded from the force plate.

Experiment 1 consisted of three experimental conditions in separated blocks. In the No Perturbation condition, a perturbation was not applied and the TA- and SOL-MEPs were measured during normal standing. In the No Cue condition, the anterior surface translation was applied suddenly without any prior information being given to the participant. In the Cue condition, an acoustic signal was provided 1.0–1.3 s before the perturbation was given as timing information. The interval between trials was randomized from 5 to 12 s in all conditions. **Figure 1** illustrates the scene of the experiment and the time course of a single trial.

To estimate the relationship between the mean MEP amplitude and the stimulus intensities, we measured the stimulus–response relation curves. Six intensities of TMS from 80% TA-MT to 130% TA-MT were chosen for each condition. The highest intensity was set to 130% TA-MT because the standing posture was disturbed in larger intensity due to TMS pulse. Each participant performed six sets with different stimulus intensities in each condition, and a single set consisted of 10 trials (6 sets × 10 trials/set). A total of 180 trials were performed (3 conditions × 60 trials/condition). The order of three experimental conditions (No Perturbation, No Cue, and Cue) and the six stimulus intensities was randomized to make the order effects equal for every participant.

#### Experiment 2

An acoustic tone by itself might have an effect on the corticospinal excitability through an arousal enhancement that is independent of postural changes (Lofberg et al., 2014). To exclude this possibility, we further examined how the TA and the SOL excitability would be modulated with an acoustic cue preceding TMS without postural perturbation. In Experiment 2, we compared stimulus–response relation curves with and without temporal cues (No Perturbation with Cue and No Perturbation, respectively). All MEPs were induced in normal standing, and no perturbation was applied. The participants performed six blocks with different stimulus intensities (80–130% TA-MT) the same as in Experiment 1. Ten trials were performed for every set, and the orders of conditions and stimulus intensities were randomized to avoid order effects.

### Data Analysis

The peak-to-peak amplitude of each MEP from all recorded data was calculated within 40 ms after the stimulus onset. All MEPs were normalized by the M-max of each muscle. We plotted the mean MEP amplitude relative to the stimulus intensities in all three conditions and fitted them to data points using linear regression analysis. We assessed the slopes of the linear regression as a gain parameter of each stimulus–response relation curve.

We first performed the Jarque–Bera test to check the normality of the MEP distribution at each stimulus intensity, and we observed that 4.0% of the TA-MEP data and 11.0% of the SOL-MEP data were identified as outliers in Experiment 1. We therefore excluded outliers with the non-parametric method, in which the discarded data were defined as deviating more than 1.5 × the interquartile range (IQR) from the 75% quartile and below 1.5 × the IQR from the 25% quartile (Papegaaij et al., 2014). Additionally, the first trial in every block was discarded from the subsequent analysis because it was known that this MEP tended to be higher compared to those in subsequent trials and that the postural response was influenced by a first trial effect (Allum et al., 2011).

We computed the pre-stimulus root mean square of the EMG activity (BGA) for 50 ms before the TMS. Trials were also discarded when the BGA exceeded the mean value plus two times its standard deviation (BGA + 2SD). In Experiment 1, we further calculated the relative difference of the MEP amplitude (MEP ratio) between the No Cue and Cue conditions. To compare the changes from the No Perturbation condition, we normalized the MEP amplitude in the No Cue and Cue conditions by the mean amplitude in the No Perturbation condition.

We compared the slopes of the response–stimulus relation curves among different conditions with a one-way analysis of variance (ANOVA) with repeated measures. For Experiment 2, we performed *t*-tests to determine whether the slopes showed significant differences between the No Perturbation with Cue and No Perturbation conditions. Both the MEP ratio and BGA were compared with two-way repeated-measures ANOVA with conditions and stimulus intensities. When the sphericity could not be assumed, a Greenhouse-Geisser correction was applied. In cases with a significant effect, we used Bonferroni correction for *post hoc* comparisons to assess the differences among conditions. The significance value for all conditions was set at *p* < 0.05, and the averaged data are presented as the mean ± standard error of the mean (SE). Interaction effects are reported only when significant.

## Results

### Experiment 1: Effects of the Predictions of Postural Perturbation on the Excitability of the Corticospinal Pathway in the TA and SOL Muscles

**FIGURE 2:**
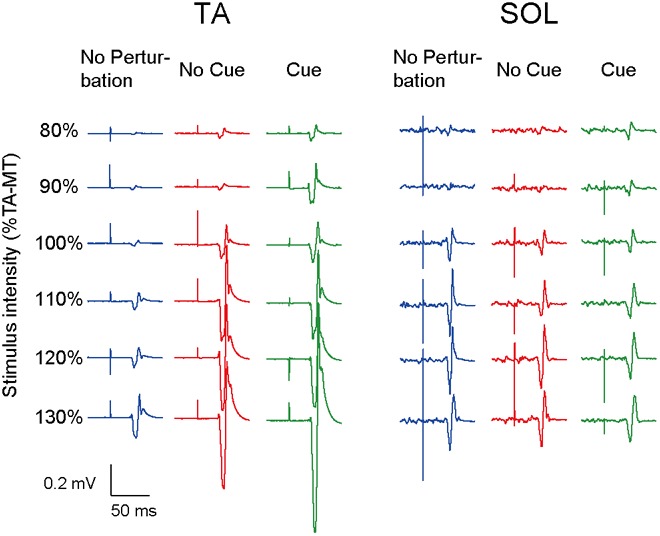
The ensemble-averaged MEPs of a representative participant in Experiment 1. Note that the TA-MEP is larger in the No Cue and Cue conditions compared to that in the No Perturbation condition, whereas the SOL-MEP is almost equal among the four conditions. TA-MT, the motor threshold of the MEP in the TA; No Perturbation, normal standing without any perturbations; No Cue, exposing perturbations without timing cue; Cue, exposing perturbations with timing cue.

**FIGURE 3:**
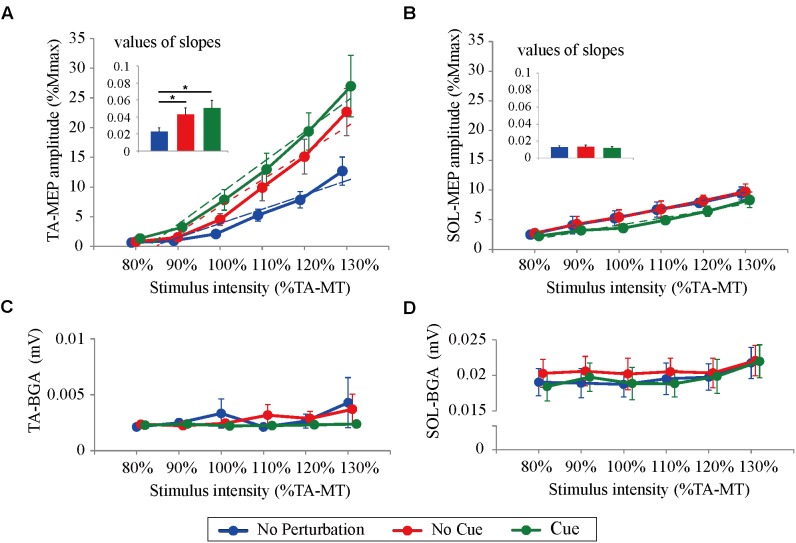
Response–stimulus relation curves of the TA- and SOL-MEP and BGA activities in each stimulus condition (80% TA-MT–130% TA-MT). Dotted lines represent regression lines in each curve. The values of MEPs were normalized by M-max. Each point is presented as the mean ± SEM. Bars represent values of slopes fitted by linear regression (^∗^*p* < 0.05 between conditions). For the TA-MEP **(A)**, the mean amplitude above 100% of the stimulus intensity and the values of the slopes are larger in the No Cue and Cue conditions compared to those in the No Perturbation condition. For the SOL-MEP **(B)**, those parameters tended to be smaller in the Cue condition than in any other condition. The BGA is similar among all conditions, both in the TA **(C)** and the SOL **(D)**. No Perturbation, normal standing without any perturbations; No Cue, exposing perturbations without timing cue; Cue, exposing perturbations with timing cue.

**FIGURE 4:**
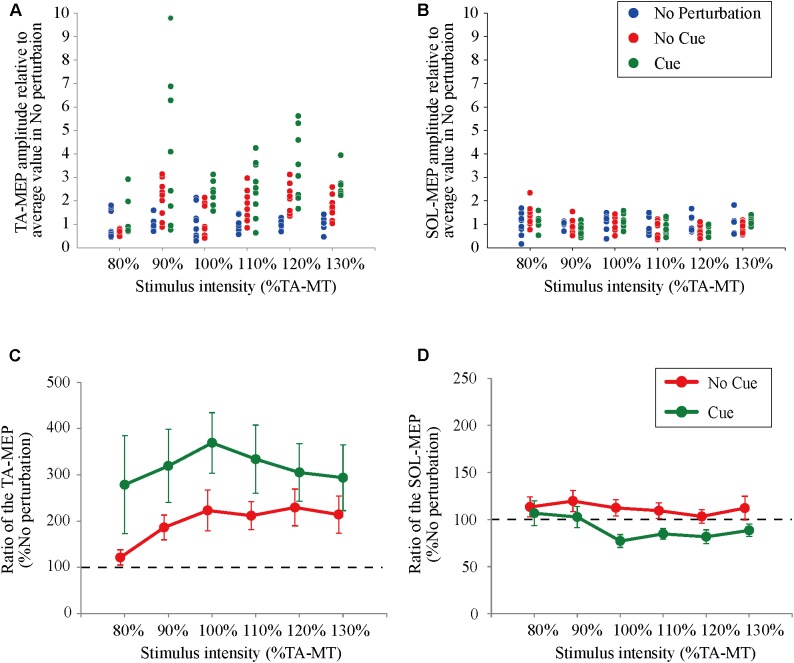
The ratio of changes in the TA- and the SOL-MEP from the mean amplitude in the No Perturbation condition. Each point represents the value of MEP in all trials divided by the mean amplitude in the No Perturbation condition for a representative subject (**A**: TA-MEP and **B**: SOL-MEP). The mean values of the MEP ratio in all subjects. “100%” corresponds to the averaged values in the No Perturbation condition of each stimulus intensity (**C**: TA-MEP ratio and **D**: SOL-MEP ratio). No Perturbation, normal standing without any perturbations; No Cue, exposing perturbations without timing cue; Cue, exposing perturbations with timing cue.

All participants completed Experiment 1 without taking any steps or falling, and all stimulus–response curves were obtained for the TA and the SOL muscles. **Figure 2** shows the ensemble-averaged MEPs of a representative participant in Experiment 1. In the No Cue and Cue conditions, the TA-MEPs were clearly enhanced compared to those in the No Perturbation condition, whereas no significant difference was found in the SOL-MEPs. In the averaged group data, the one-way repeated-measures ANOVA demonstrated a significant main effect of condition on the slopes of the TA-MEP (*F*_(1.2,17.1)_ = 11.9, *p* = 0.01, **Figure 3**). The subsequent *post hoc* test revealed that the slopes of the TA-MEP in the No Cue and Cue conditions were both significantly larger than that in the No Perturbation condition (No Perturbation 0.023 ± 0.004 vs. No Cue 0.042 ± 0.007, *p* = 0.01; No Perturbation 0.023 ± 0.004 vs. Cue 0.050 ± 0.009, *p* = 0.01), whereas no significant difference was observed in the slope of the TA-MEP between the No Cue and Cue conditions (No Cue vs. Cue, *p* = 0.14). The rates of discarded data were 6.2% of the TA-MEPs and 5.3% of the SOL-MEPs. In contrast, the slopes of the SOL-MEPs did not change significantly (*F*_(1.3,18.1)_ = 0.31, *p* = 0.83). The linear regression fitting to the stimulus–response relation curve showed high coefficients of determination (TA: mean *R*^2^ = 0.86 [0.65–0.97], SOL: mean *R*^2^ = 0.82 [0.56–0.98]). There was a significant effect of condition on the MEP ratio revealed by the two-way repeated-measures ANOVA in Experiment 1 (TA-MEP conditions: *F*_(1,13)_ = 6.31, *p* = 0.03; stimulus intensities: *F*_(2.9,38.3)_ = 0.99, *p* = 0.38; SOL-MEP conditions: *F*_(1,13)_ = 12.5, *p* = 0.004; intensities: *F*_(5,65)_ = 1.02, *p* = 0.41, **Figure 4**). The ratio of the TA-MEP in the Cue condition was significantly larger than that in No Cue condition (Cue vs. No Cue, *p* = 0.03), whereas that of the SOL-MEP in the Cue condition was significantly reduced compared to that in the No Cue condition (Cue vs. No Cue, *p* = 0.004).

There were no significant differences in the BGA in the TA muscle among the different conditions and stimulus intensities (conditions: *F*_(1.1,13.0)_ = 0.56, *p* = 0.47; stimulus intensities: *F*_(2.1,13.0)_ = 1.03, *p* = 0.33; the averaged value of all trials: No Perturbation, 2.83 ± 0.34 μV; No Cue, 2.80 ± 0.23 μV; Cue, 2.30 ± 0.29 μV). The two-way repeated-measures ANOVA showed a significant main effect of stimulus intensity on the BGA of the SOL (*F*_(5.0,65.0)_ = 3.41, *p* < 0.05; No Perturbation: 19.62 ± 0.46 μV; No Cue: 23.68 ± 0.29 μV; Cue: 19.62 ± 0.52 μV). Because no significant difference was found related to the condition (*F*_(2.0,26.0)_ = 0.73, *p* = 0.49), we concluded that the difference in the BGA in the SOL was unlikely to have caused any MEP modulation.

### Experiment 2: Effects of Temporal Prediction on TMS While Standing

**FIGURE 5:**
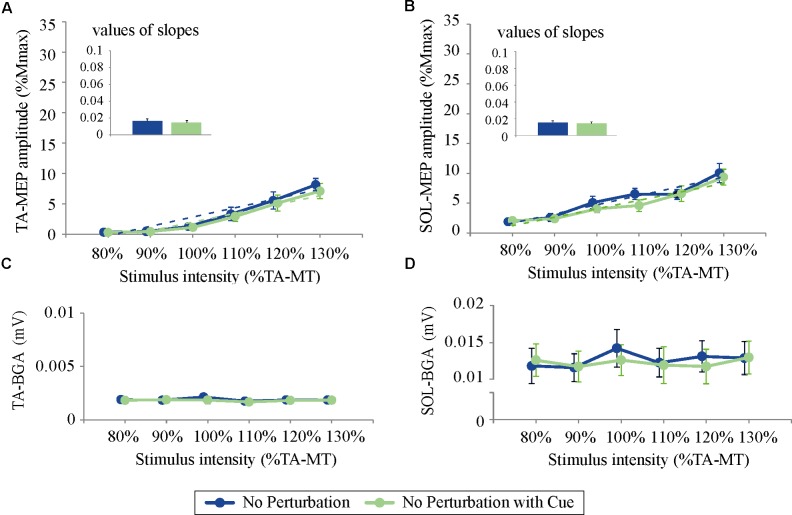
The response–stimulus relation curves of the TA- and SOL-MEP and BGA activities in Experiment 2 (80% TA-MT–130% TA-MT). Each point is presented as the mean ± SEM. Bars represent the values of the slopes fitted by linear regression. The values of MEPs were normalized by M-max. For both the TA- **(A)** and the SOL-MEP **(B)**, the mean amplitude and the values of the slopes are not different between the two conditions. The BGA is also almost the same in the two conditions (**C**: TA, **D**: SOL). No Perturbation, normal standing without any perturbations; No Perturbation with Cue, normal standing without any perturbations but with cue.

For both the TA- and SOL-MEP amplitudes, the two-way repeated-measures ANOVA did not reveal significant differences between the No Perturbation and No Perturbation with Cue conditions (TA-MEP conditions: *F*_(1,5)_ = 0.84, *p* = 0.40; stimulus intensities: *F*_(1.5,7.3)_ = 1.25, *p* = 0.32; SOL conditions: *F*_(1,5)_ = 0.53, *p* = 0.50; stimulus intensities: *F*_(5,25)_ = 1.28, *p* = 0.30, **Figure 5**). The rates of discarded data were 4.4% of the TA-MEPs and 4.7% of the SOL-MEPs. In Experiment 2, the BGA of both the TA and the SOL was not significantly different between conditions (TA conditions: *F*_(1,5)_ = 1.37, *p* = 0.30; stimulus intensities: *F*_(1.5,7.5)_ = 21.30, *p* = 0.001; No Perturbation: 1.90 ± 0.05 μV; No Perturbation with Cue: 1.82 ± 0.03 μV; SOL conditions: *F*_(1,5)_ = 4.64, *p* = 0.08; stimulus intensities: *F*_(5,25)_ = 29.03, *p* < 0.001; No Perturbation: 12.66 ± 0.40 μV; No Perturbation with Cue: 12.27 ± 0.22 μV).

## Discussion

Subliminal enhancements of the corticospinal excitability have been reported in the TA and the SOL when the subject’s posture was exposed to the more unstable environment (Solopova et al., 2003; Tokuno et al., 2009). In the present study, we observed that the TA-MEP was enhanced when the subjects knew about an upcoming perturbation compared to when they did not know, and the difference was more apparent when a timing cue was given. On the other hand, the SOL-MEP tended to be decreased slightly when a timing cue was given, whereas it was no significantly different between the conditions when the subjects knew of the upcoming perturbation versus when they did not know. These results suggest that excitability of the corticospinal pathway, especially in the ankle dorsiflexor muscle, is modulated according to not only the current posture but also the future state.

### Implication of Enhanced Corticospinal Excitability in the TA before Perturbation

The corticospinal excitability in the TA has been reported to be enhanced when walking or standing posture is exposed to more unstable conditions (Christensen et al., 2001; Obata et al., 2009; Baudry et al., 2015). In walking, the amplitude of TA-MEP was shown to be larger in the stance phase than in the swing phase (Christensen et al., 2001). Notably, the amplitude of the stretch reflex was also increased in the stance phase in which the ankle joint was likely to be unstable. Similarly, the corticospinal excitability and the stretch reflex were enhanced in standing compared to in a supine posture (Nakazawa et al., 2003; Obata et al., 2009; Baudry et al., 2015). From the viewpoint of functional significance, these modulations could be related to a safeguarding of the ankle joint stability through the facilitation of the long-latency EMG response. Since this reflex is partially mediated by the transcortical pathway, an enhanced excitability of the corticospinal pathway would amplify the reflex response. Therefore, the present finding of enhanced TA-MEP before perturbation may reflect a presetting of the EMG response at a higher level when perturbation is expected. In regard to the mechanisms of this enhancement, the autonomic response is one of the candidates in addition to prediction. It is well known that fear, unpleasant emotions, and elevated arousal can enhance corticospinal excitability (Baumgartner et al., 2007; Hajcak et al., 2007; Giovannelli et al., 2013). Cortical and subcortical routes through the emotion-processing regions, such as the amygdala and the anterior cingulate cortex, are regarded as primary sources for these modulations (Grezes et al., 2007). If postural perturbations induce changes in such psychological states, corticospinal excitability might be altered regardless of predictions. To explore this possibility in greater detail, future studies should pay attention to the relationship between these excitability and autonomic responses, such as the skin conductance response, heart rate, and pupil diameter.

### The Effect of Temporal Prediction on Corticospinal Excitability in the TA

Electroencephalography (EEG) studies have demonstrated that temporal prediction of the onset of postural perturbation changes the cortical activities in the motor-related area (Jacobs et al., 2008; Mochizuki et al., 2009). Jacobs et al. (2008) reported that the contingent negative variation at Cz and Pz was modulated by a timing cue in the preparatory period for a perturbation, and the extent of modulation was correlated with the COP displacement. Our present observations of the corticospinal pathways support those findings, in that a preceding auditory cue affects the state of the motor area.

As a neural basis of this modulation in the motor area, the cerebellum is thought to be a key region. The cerebellum has been shown to contribute to the recalibration of the estimated timing for a moving target to disappear temporally (Roth et al., 2013). In fact, cerebellar patients are known to have disrupted temporal aspects of their upper limb movements, as occurs with an adjustment of anticipatory muscle activities (Lang and Bastian, 1999) and the sequence of the long-latency EMG response (Horak and Diener, 1994; Jacobs et al., 2008). Considering that TMS of the cerebellum suppresses the motor cortex through cerebellar-motor connectivity (Ugawa et al., 1995; Pinto and Chen, 2001), it is conceivable that projections from the cerebellum affect the modulations of corticospinal excitability with the temporal prediction.

Our present analyses demonstrated that the corticospinal excitability in the TA was enhanced when the timing cue was given. This result is inconsistent with previous studies of the stretch reflex that reported the reduced amplitude of the long-latency response (Nakazawa et al., 2009; Fujio et al., 2016). We suspect that this discrepancy is due to the difference in perturbation types. In those experiments, the stretch reflex in the TA was evoked by rotation of the ankle joint (i.e., plantar flexion) or landing from a low stand in which the TA response is not required in order to maintain the standing posture. On the contrary, the TA response evoked by an anterior-surface translation contributes to recovery from the posterior sway. Therefore, our observation of enhanced TA-MEP, which would enhance the TA response, is reasonable with respect to the context for the postural recovery.

### The Different Changes in Corticospinal Excitability between the TA and the SOL

Unlike the pronounced facilitations in the TA muscle, the SOL-MEP was slightly reduced only when the timing cue was provided. This result is consistent with a previous report (Walchli et al., 2017). We propose that this discrepancy is related to differences in the functional and physiological features of these muscles.

First, as mentioned above, the manner of perturbation used in this study should be taken into consideration. Types and directions of perturbation are determinants for EMG responses (Moore et al., 1988). When anterior surface translation is applied, the TA activation is evoked predominantly to compensate for posterior body sway, whereas the SOL activation is likely to deteriorate posterior-swayed posture rather than compensate for it. We suspect that if the modulation observed before perturbation is related to the postural response, the subliminal enhancement of TA-MEP would be reasonable for the selective activation of the TA. In this regard, Walchli et al. (2017) reported that the SOL-MEP was not modulated before posterior translation was applied. In this case, the SOL activity is thought to contribute to postural recovery from anterior body sway. It suggests that the manner of perturbation has little influence on the difference between the TA and the SOL.

Second, the susceptibility of the corticospinal pathway to the TMS could be a reason explaining the difference between the TA and the SOL. It has been reported that the MEP of the TA was more easily elicited than that of the SOL, in both sitting and supine postures (Brouwer and Ashby, 1990; Lavoie et al., 1995; Bawa et al., 2002; Obata et al., 2009). The inhibitory projection of the corticospinal pathway to spinal motoneurons in the SOL was reported to be richer than that in the TA (Hudson et al., 2013). The inconsistency between muscles may be attributable to such different proportions of the excitatory and inhibitory projections. In support of this, review by Dietz (1992) suggested that the SOL is more influenced by spinal proprioceptive feedback whereas cortical control is dominant in the TA during landing and standing.

Third, the reciprocal inhibition could involve the slight reduction of the SOL excitability with temporal prediction. It has been known that the motoneurons of the TA and the SOL receive input from interneurons of the reciprocal inhibition, which are driven by the motor cortex through the corticospinal pathway (Geertsen et al., 2010). The ratio of the enhancement in the prime mover was larger relative to that of the inhibition in the antagonist muscles (Gerachshenko and Stinear, 2007). This is consistent with our present finding that the reduction of the SOL excitability was observed only when the larger TA-MEPs were elicited with temporal prediction.

### Limitations

There are some limitations regarding the interpretation of the present results. First, the site and the intensity of the TMS were adjusted for the TA but not for the SOL. Although the hot spot of the SOL is thought to be almost the same as that of the TA, we cannot exclude the possibility that the subtle difference of the stimulus site might affect the results in the SOL. We observed little changes of the SOL-MEP despite the application of wide range of stimulus intensities. However, it is still possible that the TMS intensity relative to the motor threshold of the TA masked the modulation in the SOL. Second, we used one perturbation through experiment because we focused on the temporal prediction only in this study. It is still unclear about the effects of predictions on direction and intensity of perturbation. Further studies are needed to clarify in this regard. Third, as mentioned above, we could not divide the influence of autonomic response from the effects of prediction. In order to test our results more rigorously, other measurements to assess the dynamics of the autonomic nervous system must be added to the experimental protocol.

## Conclusion

We sought to clarify the effects of the prediction of postural perturbation on the excitability of corticospinal pathways in the TA and SOL muscles. We observed that TA excitability was enhanced just before perturbation. Specifically, when a timing cue was provided, the enhancement of the TA excitability was more remarkable, whereas the SOL excitability was slightly reduced. These results imply that prediction of the postural state is one modulator of the excitability of corticospinal pathways. We propose that TA excitability is sensitive to potential risks to posture security and that an accurate temporal preparation is available to preset the TA excitability to upcoming perturbation.

## Author Contributions

KF carried out the experiments and wrote the manuscript with support from HO and KN. HO, TK, and NK helped to decide on the experimental protocol. KN supervised the project.

## Conflict of Interest Statement

The authors declare that the research was conducted in the absence of any commercial or financial relationships that could be construed as a potential conflict of interest.

## References

[B1] Allum J. H. Tang K. S. Carpenter M. G. Oude Nijhuis L. B. Bloem B. R. (2011). Review of first trial responses in balance control: influence of vestibular loss and Parkinson’s disease. *Hum. Mov. Sci.* 30 279–295. 10.1016/j.humov.2010.11.009 21435732

[B2] Baudry S. Collignon S. Duchateau J. (2015). Influence of age and posture on spinal and corticospinal excitability. *Exp. Gerontol.* 69 62–69. 10.1016/j.exger.2015.06.006 26055449

[B3] Baumgartner T. Willi M. Jancke L. (2007). Modulation of corticospinal activity by strong emotions evoked by pictures and classical music: a transcranial magnetic stimulation study. *Neuroreport* 18 261–265. 10.1097/WNR.0b013e328012272e 17314668

[B4] Bawa P. Chalmers G. R. Stewart H. Eisen A. A. (2002). Responses of ankle extensor and flexor motoneurons to transcranial magnetic stimulation. *J. Neurophysiol.* 88 124–132. 10.1152/jn.2002.88.1.124 12091538

[B5] Brouwer B. Ashby P. (1990). Corticospinal projections to upper and lower limb spinal motoneurons in man. *Electroencephalogr. Clin. Neurophysiol.* 76 509–519. 10.1016/0013-4694(90)90002-2 1701119

[B6] Christensen L. O. Andersen J. B. Sinkjaer T. Nielsen J. (2001). Transcranial magnetic stimulation and stretch reflexes in the tibialis anterior muscle during human walking. *J. Physiol.* 531 545–557. 10.1111/j.1469-7793.2001.0545i.x 11230526 PMC2278473

[B7] Dietz V. (1992). Human neuronal control of automatic functional movements: interaction between central programs and afferent input. *Physiol. Rev.* 72 33–69. 10.1152/physrev.1992.72.1.33 1731372

[B8] Fujio K. Obata H. Kawashima N. Nakazawa K. (2016). The effects of temporal and spatial predictions on stretch reflexes of ankle flexor and extensor muscles while standing. *PLoS One* 11:e0158721. 10.1371/journal.pone.0158721 27385043 PMC4934788

[B9] Geertsen S. S. Zuur A. T. Nielsen J. B. (2010). Voluntary activation of ankle muscles is accompanied by subcortical facilitation of their antagonists. *J. Physiol.* 588 2391–2402. 10.1113/jphysiol.2010.190678 20457734 PMC2915515

[B10] Gerachshenko T. Stinear J. W. (2007). Suppression of motor evoked potentials in biceps brachii preceding pronator contraction. *Exp. Brain Res.* 183 531–539. 10.1007/s00221-007-1071-4 17665175

[B11] Giovannelli F. Banfi C. Borgheresi A. Fiori E. Innocenti I. Rossi S. (2013). The effect of music on corticospinal excitability is related to the perceived emotion: a transcranial magnetic stimulation study. *Cortex* 49 702–710. 10.1016/j.cortex.2012.01.013 22405960

[B12] Grezes J. Pichon S. De Gelder B. (2007). Perceiving fear in dynamic body expressions. *Neuroimage* 35 959–967. 10.1016/j.neuroimage.2006.11.030 17270466

[B13] Hajcak G. Molnar C. George M. S. Bolger K. Koola J. Nahas Z. (2007). Emotion facilitates action: a transcranial magnetic stimulation study of motor cortex excitability during picture viewing. *Psychophysiology* 44 91–97. 10.1111/j.1469-8986.2006.00487.x 17241144

[B14] Horak F. B. Diener H. C. (1994). Cerebellar control of postural scaling and central set in stance. *J. Neurophysiol.* 72 479–493. 10.1152/jn.1994.72.2.479 7983513

[B15] Horak F. B. Diener H. C. Nashner L. M. (1989). Influence of central set on human postural responses. *J. Neurophysiol.* 62 841–853. 10.1152/jn.1989.62.4.841 2809706

[B16] Hudson H. M. Griffin D. M. Belhaj-Saif A. Cheney P. D. (2013). Cortical output to fast and slow muscles of the ankle in the rhesus macaque. *Front. Neural Circuits* 7:33. 10.3389/fncir.2013.00033 23459919 PMC3585439

[B17] Jacobs J. V. Fujiwara K. Tomita H. Furune N. Kunita K. Horak F. B. (2008). Changes in the activity of the cerebral cortex relate to postural response modification when warned of a perturbation. *Clin. Neurophysiol.* 119 1431–1442. 10.1016/j.clinph.2008.02.015 18397840 PMC2443739

[B18] Lang C. E. Bastian A. J. (1999). Cerebellar subjects show impaired adaptation of anticipatory EMG during catching. *J. Neurophysiol.* 82 2108–2119. 10.1152/jn.1999.82.5.2108 10561391

[B19] Lavoie B. A. Cody F. W. Capaday C. (1995). Cortical control of human soleus muscle during volitional and postural activities studied using focal magnetic stimulation. *Exp. Brain Res.* 103 97–107. 10.1007/BF00241968 7615042

[B20] Lofberg O. Julkunen P. Paakkonen A. Karhu J. (2014). The auditory-evoked arousal modulates motor cortex excitability. *Neuroscience* 274 403–408. 10.1016/j.neuroscience.2014.05.060 24928350

[B21] Mochizuki G. Sibley K. M. Cheung H. J. Mcilroy W. E. (2009). Cortical activity prior to predictable postural instability: is there a difference between self-initiated and externally-initiated perturbations? *Brain Res.* 1279 29–36. 10.1016/j.brainres.2009.04.050 19422812

[B22] Moore S. P. Rushmer D. S. Windus S. L. Nashner L. M. (1988). Human automatic postural responses: responses to horizontal perturbations of stance in multiple directions. *Exp. Brain Res.* 73 648–658. 10.1007/BF00406624 3224674

[B23] Nakazawa K. Kawashima N. Akai M. (2009). Effect of different preparatory states on the reflex responses of ankle flexor and extensor muscles to a sudden drop of support surface during standing in humans. *J. Electromyogr. Kinesiol.* 19 782–788. 10.1016/j.jelekin.2008.01.002 18304836

[B24] Nakazawa K. Kawashima N. Obata H. Yamanaka K. Nozaki D. Akai M. (2003). Facilitation of both stretch reflex and corticospinal pathways of the tibialis anterior muscle during standing in humans. *Neurosci. Lett.* 338 53–56. 10.1016/S0304-3940(02)01353-8 12565139

[B25] Obata H. Sekiguchi H. Nakazawa K. Ohtsuki T. (2009). Enhanced excitability of the corticospinal pathway of the ankle extensor and flexor muscles during standing in humans. *Exp. Brain Res.* 197 207–213. 10.1007/s00221-009-1874-6 19603153

[B26] Papegaaij S. Taube W. Hogenhout M. Baudry S. Hortobagyi T. (2014). Age-related decrease in motor cortical inhibition during standing under different sensory conditions. *Front. Aging Neurosci.* 6:126. 10.3389/fnagi.2014.00126 24971063 PMC4054792

[B27] Petersen N. Christensen L. O. Morita H. Sinkjaer T. Nielsen J. (1998). Evidence that a transcortical pathway contributes to stretch reflexes in the tibialis anterior muscle in man. *J. Physiol.* 512(Pt 1), 267–276. 10.1111/j.1469-7793.1998.267bf.x 9729635 PMC2231172

[B28] Pinto A. D. Chen R. (2001). Suppression of the motor cortex by magnetic stimulation of the cerebellum. *Exp. Brain Res.* 140 505–510. 10.1007/s002210100862 11685404

[B29] Roth M. J. Synofzik M. Lindner A. (2013). The cerebellum optimizes perceptual predictions about external sensory events. *Curr. Biol.* 23 930–935. 10.1016/j.cub.2013.04.027 23664970

[B30] Solopova I. A. Kazennikov O. V. Deniskina N. B. Levik Y. S. Ivanenko Y. P. (2003). Postural instability enhances motor responses to transcranial magnetic stimulation in humans. *Neurosci. Lett.* 337 25–28. 10.1016/S0304-3940(02)01297-1 12524163

[B31] Taube W. Schubert M. Gruber M. Beck S. Faist M. Gollhofer A. (2006). Direct corticospinal pathways contribute to neuromuscular control of perturbed stance. *J. Appl. Physiol.* 101 420–429. 10.1152/japplphysiol.01447.2005 16601305

[B32] Tokuno C. D. Taube W. Cresswell A. G. (2009). An enhanced level of motor cortical excitability during the control of human standing. *Acta Physiol.* 195 385–395. 10.1111/j.1748-1716.2008.01898.x 18774948

[B33] Ugawa Y. Uesaka Y. Terao Y. Hanajima R. Kanazawa I. (1995). Magnetic stimulation over the cerebellum in humans. *Ann. Neurol.* 37 703–713. 10.1002/ana.410370603 7778843

[B34] Walchli M. Tokuno C. D. Ruffieux J. Keller M. Taube W. (2017). Preparatory cortical and spinal settings to counteract anticipated and non-anticipated perturbations. *Neuroscience* 365 12–22. 10.1016/j.neuroscience.2017.09.032 28951323

